# Epidemiology, Outcomes, and Trend Analysis of Hospitalized Infants With Respiratory Syncytial Virus (RSV) Bronchiolitis From 1997 to 2019

**DOI:** 10.7759/cureus.64229

**Published:** 2024-07-10

**Authors:** Daniel Torres, Pooja Musuku, Prithvi Sendi, Balagangadhar R Totapally

**Affiliations:** 1 Pediatric Critical Care Medicine, Nicklaus Children's Hospital, Miami, USA; 2 Pediatrics, Herbert Wertheim College of Medicine, Miami, USA

**Keywords:** epidemiology, child, complex chronic condition, respiratory syncytial virus (rsv), national trend

## Abstract

Background

Most children with respiratory syncytial virus (RSV) infection have a self-limiting course that can be managed with supportive care, and hospitalization is uncommon. The objectives of this study were to evaluate the epidemiology, outcomes, associated comorbidities, and temporal trends in the prevalence of infants one to 24 months of age who required hospitalization for RSV infection in the United States of America from 1997 to 2019.

Methods

In this retrospective cross-sectional study, we utilized the Kids' Inpatient Database (KID) to investigate the prevalence and outcomes of RSV bronchiolitis within a large cohort of discharged patients from 1997 to 2019. We included children one to 24 months of age admitted with a diagnosis of RSV bronchiolitis. Neonates were excluded from the analysis. A chi-square for linear trend was used to analyze trends in the prevalence of RSV bronchiolitis hospitalization, the presence of complex chronic conditions (CCC), congenital heart disease (CHD), the use of non-invasive and invasive mechanical ventilation (NIV and IMV), and hospital mortality.

Results

There were a total of 566,786 infants aged one to 24 months hospitalized with RSV infection out of a total of 9,309,597 discharges during the eight-year cohort, with a hospital prevalence of 60.9 per 1000 discharges and a hospital mortality rate of 0.09% (95% confidence interval (CI): 0.08%-0.1%). There was no trend in hospitalization rates of RSV infections per 100,000 U.S. population during the study period, with a decrease in hospital mortality trend. Children with RSV bronchiolitis were more likely to have government insurance and reside in zip codes with the lowest income quartile. There was a significant seasonal and regional variation in RSV-related hospitalizations. The presence of CCC was identified in 2.4% of the RSV group compared to 5.1% of non-RSV discharges (odds ratio (OR): 0.46, 95% CI: 0.45-0.47; p<0.001). The prevalence of RSV among all discharges has significantly increased over the study period, rising from 51.6 cases per 1000 discharges in 1997 to 180.1 cases per 1000 discharges in 2019 (p<0.001). The prevalence of CCC and CHD among RSV patients has also shown an upward trend, with CCC cases increasing from 1,411 in 1997 to 2,795 in 2019 and CHD cases rising from 1,795 to 3,622 during the same period. The use of invasive mechanical ventilation, non-invasive ventilation, and extracorporeal membrane oxygenation has consistently increased over time. Additionally, complications such as the need for cardiopulmonary resuscitation have demonstrated a similar increasing trend, although they have remained overall low. However, population-based hospitalization rates showed no significant trend.

Conclusions

The hospitalization rates at a population level in the United States for RSV infection in children aged one to 24 months remained steady from 1997 to 2019, while hospital mortality rates showed a declining trend. There is an increased proportion of comorbid conditions and increased resource utilization in children with RSV. These findings are important for monitoring the effectiveness of preventive strategies for severe RSV infections.

## Introduction

Bronchiolitis is a common lower respiratory tract infection in infants and young children, and respiratory syncytial virus (RSV) is the most common cause of bronchiolitis. RSV bronchiolitis typically affects children in the first two years of life [1]. There are seasonal differences in the incidence of RSV infection in the United States, with the highest incidence occurring from December to March [1].

Nearly all children will get infected with RSV at least once by the time they reach two years of age [1]. Most children have a self-limited course that responds to supportive home care; however, it is estimated that more than 57,500 hospitalizations and 2.1 million outpatient visits are associated with RSV infections each year in U.S. children younger than five years [1].

Globally, RSV is estimated to cause 3.2 million hospitalizations and 118,000 deaths in children younger than five years. In the United States, RSV-attributable mortality is low and is typically associated with underlying complex medical conditions. However, RSV infections' direct and indirect costs are vast and likely underestimated [2]. Nosocomial transmission has been reported for several decades and is particularly problematic in vulnerable patient populations, such as patients in pediatric wards or patients with immunocompromised conditions [2].

Several studies in RSV-infected children have investigated risk factors associated with a more severe clinical course. Younger age at presentation, lower weight on admission, prematurity, early ventilatory support, associated congenital heart disease, chronic lung disease, immunodeficiency, specific neuroendocrine profile, specific polymorphisms, and elevated liver transaminases have all been associated with a longer duration of mechanical ventilation in RSV-infected children [3]. Known risk factors for RSV infection include lack of breastfeeding, passive smoking exposure, attendance at childcare, school-aged siblings in the home, overcrowded housing, and a lack of caregiver health literacy [4]. The mortality rate is higher in premature babies and low-birth-weight babies [5].

Hospital management for severe RSV infections focuses on supportive care, which includes correcting hypoxemia, appropriate hydration and feeding, airway suctioning, and sometimes other respiratory support, including invasive and non-invasive mechanical ventilation [4].

Severe infection in infants can be prevented by vaccinating pregnant women or administering antibodies to children after birth [6]. Monoclonal antibodies have been found to have substantial benefits in the prevention of RSV infection in infants [7]. To prevent severe RSV disease in infants, the Centers for Disease Control and Prevention recommend either maternal RSV vaccination (one dose during weeks 32 through 36 of pregnancy) or infant immunization with RSV monoclonal antibodies (nirsevimab) [8]. Palivizumab is recommended for high-risk children for the prevention of RSV infection [9,10]. The choice of monoclonal antibody may change with the availability of medications and efficacy data.

The recent epidemiological trends at a national level regarding the prevalence of hospitalization, comorbidity, and outcomes are essential to assessing the effectiveness of ongoing public health measures such as immunoprophylaxis. The objectives of this study were to evaluate the outcomes, associated comorbidities, and temporal trends in the prevalence of infants one to 24 months of age who required hospitalization for RSV infection in the United States of America from 1997 to 2019.

## Materials and methods

The study was conducted at Nicklaus Children's Hospital, Miami, FL, USA. We performed a retrospective cross-sectional analysis of the Kids' Inpatient Database (KID) from 1997 to 2019 for discharged patients. The KID is one of a family of databases and software tools developed as part of the Healthcare Cost and Utilization Project (HCUP). It was explicitly designed to permit researchers to study a broad range of conditions and procedures related to child health issues. The data included were from 1997, 2000, 2003, 2006, 2009, 2012, 2016, and 2019.

We reviewed all reported cases of RSV bronchiolitis based on the International Classification of Diseases (ICD) diagnosis codes 466.11 (ICD-9) and J21.0 (ICD-10). We included patients aged one month to 24 months. Neonates were excluded from this study.

Statistical analysis

The prevalence of RSV bronchiolitis is reported per 1000 discharges in the age group of one month to 24 months. The hospital mortality rate is reported per 100 discharges with an RSV diagnosis. To calculate the rate of hospitalization at a population level, we used a mid-year age-specific population from the U.S. Census Bureau (https://wonder.cdc.gov/bridged-race-population.html). Hospitalization rates per population are presented per 100,000 mid-year age-specific population.

A chi-square for linear trend (StatCalc of Epi Info 7, CDC, Atlanta, GA) was used to analyze trends in the prevalence of RSV bronchiolitis hospitalization, the presence of complex chronic conditions (CCC), and hospital mortality. Pediatric CCCs were defined by the pediatric complex chronic conditions classification system version 2 based on the inpatient diagnosis and procedure codes [11]. We used the presence of respiratory, neonatal, or technology dependence in the CCC group for trend analysis. In addition, the presence of congenital heart disease (CHD) was used as a separate variable for analysis.

We compared demographic variables, interventions, complications, and outcome variables between discharges with RSV bronchiolitis and all other discharges of the same age. All categorical variables (gender, age, race, patient location, and median household income (MHI) for the zip code) were analyzed with Chi-square tests. Racial and ethnic characteristics were grouped as White, Black, Hispanic, and Other (comprising Asian/Pacific Islanders, Native American, other, and unknown race/ethnicity) children. HCUP defines MHI based on the zip code in which the child resides. The zip codes are stratified by quartiles, with 1 representing the lowest and 4 representing the highest income. Continuous variables, length of stay, and hospital charges (inflation-adjusted to 2019 values) were presented as medians and interquartile ranges (IQR) and compared using the Mann-Whitney U or Kruskall-Wallis tests.

Binary regression analyses were performed with outcome variables, non-invasive ventilation (NIV), invasive mechanical ventilation (IMV), and mortality. The independent variables included in the model included age (one to 12 months vs. 12-24 months), gender, MHI quartiles, the calendar year of admission, and the presence of CCC and CHD. Clinically relevant and statistically significant (on univariate analysis) pre-morbid predictive variables were included in the model. Among the confounding variables, one with the best-performing variable was included. For example, we have included only the income status in the model, among the demographic variables, race/ethnicity, payor status, and income status.

We calculated the Brier score to assess overall model performance because the Hosmer and Lemeshow test is very sensitive to a large sample size. We generated receiver operating curves for sensitivity analysis between predictive and actual outcomes.

Binomial data are presented as odds ratios (OR) with 95% confidence intervals (CI). P-values <0.05 are considered statistically significant. We combined the data from all eight years for binomial analyses. All data were weighted according to HCUP recommendations before analysis to calculate national estimates. The data were analyzed using IBM SPSS Statistics for Windows, Version 28 (Released 2021; IBM Corp., Armonk, New York, United States) or StatCalc of Epi Info^TM^ (Centers for Disease Control and Prevention (CDC), Atlanta, GA). The Western IRB approved this study as exempt. This article was previously presented as a meeting abstract at the 51st Critical Care Congress, April 18-21, 2022.

## Results

Demographic characteristics

There were a total of 566,786 infants aged one to 24 months hospitalized with RSV infection out of a total of 9,309,597 discharges during the eight-year cohort, with a hospital prevalence of 60.9 per 1000 discharges and a hospital mortality rate of 0.09% (95% confidence interval (CI): 0.08%-0.1%). Males comprised 57.5% of the RSV discharges and 54.0% of the total discharges (p<0.001). The racial and ethnic distribution is presented in Table 1. Children with RSV bronchiolitis were more likely to have government insurance and reside in zip codes with the lowest income quartile (Table 1). There was significant regional variation in hospitalization rates, with lower rates in the South region (Table 1). As expected, there was a significant seasonal variation in hospitalizations with RSV bronchiolitis (Table 1). CCC was identified in 2.44% (95% CI: 2.39%-2.49% ) within the RSV group compared to 5.1% of non-RSV discharges (OR: 0.46, 95% CI: 0.45-0.47; p<0.001).

**Table 1:** Demographic characteristics of children one to 24 months with RSV bronchiolitis hospitalized from 1997 – 2019 in the United States. CCC: complex chronic condition; OR: odds ratio; RSV: respiratory syncytial virus *Data is presented as % and 95% confidence intervals

Variable	Patients with RSV (n=566,786)	Patients without RSV (n=8,742,811)	Significance
Male*	57.5 (55.3-57.6)	54.0 (53.9-54.0)	OR: 1.15 (1.14-1.16)
Race/ethnicity*			
White	50.5 (50.3-50.7)	48.2 (48.1-48.3)	<0.001
Black	15.8 (15.6-15.9)	16.8 (16.8-16.9)	
Hispanic	24.9 (24.7-25.0)	26.3 (26.2-26.3)	
Others	8.9 (8.8-9.0)	8.7 (8.6-8.7)	
Payor*			
Government	57.6 (57.4-57.8)	49.2 (49.1-49.2)	<0.001
Private	36.6 (36.4-36.8)	42.8 (42.7-42.9)	
Others	8.1 (8.0-8.1)	5.8 (5.7-5.9)	
Patient region*			
Northeast	16.6 (16.4–16.7)	17.5 (17.4–17.5)	<0.001
Midwest	22.2 (22.1–22.4)	9.7 (9.6–9.7)	
South	40.3 (40.1–40.5)	61.0 (60.9–61.1)	
West	20.9 (20.8–21.1)	11.9 (11.8–11.9)	
Children's hospital*	20.7 (20.5-20.8)	12.2 (12.2-12.2)	OR: 1.82 (1.80-1.83)
Median household income*			
0-25th percentile	32.7 (32.6-32.9)	29.9 (29.8-30.0)	<0.001
26th to 50th percentile	26.5 (26.3-26.7)	25.5 (25.4-25.6)	
51st to 75th percentile	21.9 (21.8-22.1)	22.2 (22.2-22.3)	
76th to 100th percentile	18.2 (18.1-18.4)	21.4 (21.3-21.5)	
Weekend admissions*	23.7 (23.6-23.9)	18.6 (18.6-18.7)	OR: 1.25 (1.24-1.26)
Discharge quarters*			
Jan - Mar	59.9 (59.7-60.1)	27.1 (27.0–27.2)	<0.001
Apr - Jun	7.5 (7.4–7.6)	24.2 (24.1–24.2)	
Jul - Sep	2.4 (2.3–2.4)	23.7 (23.6–23.7)	
Oct - Dec	30.2 (30.0–30.4)	25.1 (25.0–25.2)	
CCC *	2.44 (2.39-2.49)	5.13 (5.11-5.15)	OR: 0.46 (0.45-0.47)
Hospital mortality*	0.09 (0.08-0.1)	0.45 (0.44-0.45)	OR: 0.19 (0.18-0.21)

Complications and outcomes

A total of 13,819 (2.4%) children were identified as having an RSV infection and CCC. Additionally, 18,237 (3.22%; 95% CI: 3.16%-3.28%) patients with RSV infection had congenital heart disease (CHD), while 11,083 (2.0%) patients required an NIV and 18,411 (3.2%) patients required an IMV. Cardiac arrest or cardiopulmonary resuscitation was documented in 0.13% (95% CI: 0.12%-0.14%) patients with RSV infection, and 0.045% (95% CI: 0.041%-0.055%) received extra corporeal membrane oxygenator (ECMO) support (Table 2).

**Table 2:** The trend in the prevalence of bronchiolitis hospitalization, resource utilization, complications, and mortality in children aged one to 24 months in the United States from 1997 to 2019. CCC: complex chronic condition; CHD: congenital heart disease; CPR: cardiopulmonary resuscitation; ECMO: extracoprporeal membrane oxygenation; IQR: interquartile range; NIV: non-invasive ventilation; * prevalence per 1000 discharges; ** prevalence per 100,000 mid-year age-specific U.S. population; #Kruskal-Wallis test

Complication	1997 (n=69,854)	2000 (n=74,744)	2003 (n=73,700)	2006 (n=78,853)	2009 (n=68,479)	2012 (n=70,770)	2016 (n=58,952)	2019 (n=71,430)	Total= 566,786	Trend analysis
Hospital prevalence*	51.60	46.80	42.20	45.70	43.60	141.0	140.9	180.1	60.9	<0.001
US prevalence**	617.31	652.51	618.55	656.99	561.97	595.45	492.92	620.25	601.46	0.54
CCC*	1,411 (1,288–1,534)	1,214 (1,105–1,323)	1,286 (1,194–1,378)	1,549 (1,448–1,650)	1,645 (1,547–1,742)	1,750 (1,652–1,848)	2,169 (2,061–2,276)	2,795 (2,674–2,915)	13, 819 (13, 520–13,520)	<0.001
CHD*	1,795 (1,653-1,937)	1,973 (1,831-2,115)	1,848 (1,736-1,959)	1,981 (1,868-2,093)	1,899 (1,794-2,003)	2,189 (2,080-2,298)	2,930 (2,806-3,056)	3,622 (3,485-3,758)	18,237 (17,891-18,582)	<0.001
Invasive ventilation*	1,693 (1,557-1,829)	2,121 (1,976-2,266)	2,097 (1,979-2,215)	2,341 (2,217-2,465)	2,045 (1,937-2,154)	2,106 (1,998-2,213)	2,765 (2,643-2,887)	3,242 (3,113-3,371)	18,411 (18,064-18,758)	<0.001
NIV*	122 (91-152)	274 (223-326)	329 (282-376)	545 (485-605)	722 (658-786)	1,118 (1,040-1,195)	2,640 (2,521-2,758)	5,333 (5,167-5,498)	11,083 (10,838-11,328)	<0.001
CPR/cardiac arrest*	61 (34-89)	59 (38-81)	64 (43-85)	56 (37-75)	89 (66-112)	79 (58-100)	138 (111-165)	187 (156-218)	734 (665-802)	<0.001
ECMO*	17 (4-30)	16 (5-28)	25 (12-38)	11 (3-20)	21 (10-33)	36 (22-50)	60 (42-78)	70 (51-89)	256 (217-295)	<0.001
LOS (days, median (IQR))	3 (2-4)	3 (2-4)	3 (2-4)	3 (2-4)	3 (2-4)	3 (2-4)	3 (2-5)	3 (2-5)	3 (2-4)	<0.001#
Charges ($, median (IQR))	7,008 (4,451-11,824)	6,506 (3,988-11,282)	7,759 (4,713-13,518)	8,726 (5,235-15,422)	10,122 (5,984-18,306)	11,290 (6,505-20,930)	15,920 (8,807-31,634)	19,168 (10,214-38,454)	9,320 (5,370-17,785)	<0.001#
Case fatality rate*	0.11 (0.08-0.17)	0.11 (0.08-0.16)	0.10 (0.07-0.13)	0.08 (0.06-0.11)	0.06 (0.04-0.09)	0.07 (0.05-0.1)	0.11 (0.9-0.15)	0.07 (0.05-0.1)	0.09 (0.08-0.1)	0.0069

Trend analyses

Hospital Prevalence

The hospital prevalence of RSV infection exhibited a significant rise, increasing from 51.6 cases per 1000 discharges in 1997 to 180.1 cases per 1000 discharges in 2019 (p<0.001). Additionally, the prevalence of CCC demonstrated an upward trend, with a total of 1,411 patients (2%) in 1997, escalating to 2,795 patients (3.9%) in 2019 (Table 2). The prevalence of CHD in RSV discharges also increased from 1997 to 2019. In addition, IMV, NIV, and ECMO utilization demonstrated a consistent upward trend throughout the study period. Similarly, cardiac arrest/cardiopulmonary resuscitation (CPR) rates exhibited a similar increasing trend. However, the case fatality rate decreased during the study period. Trend analysis data per hospital discharge are presented in Table 2.

Prevalence in U.S. Population

**Figure 1:**
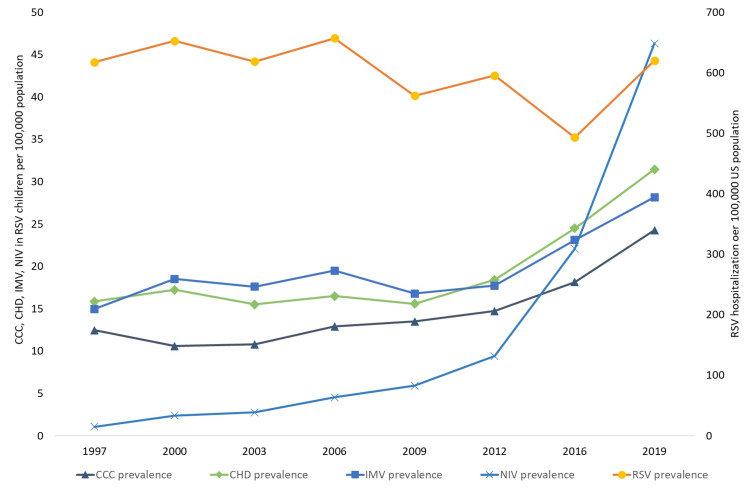
The prevalence of hospitalization with RSV infection in children aged one to 24 months per 100,000 age-specific population from 1997 to 2019 and the prevalence of complex chronic conditions, congenital heart disease, non-invasive ventilation, and invasive mechanical ventilation per 100,000 population among children with RSV infection. CCC: complex chronic conditions; CHD: congenital heart disease; IMV: invasive mechanical ventilation; NIV: non-invasive ventilation; RSV: respiratory syncytial virus

**Figure 2:**
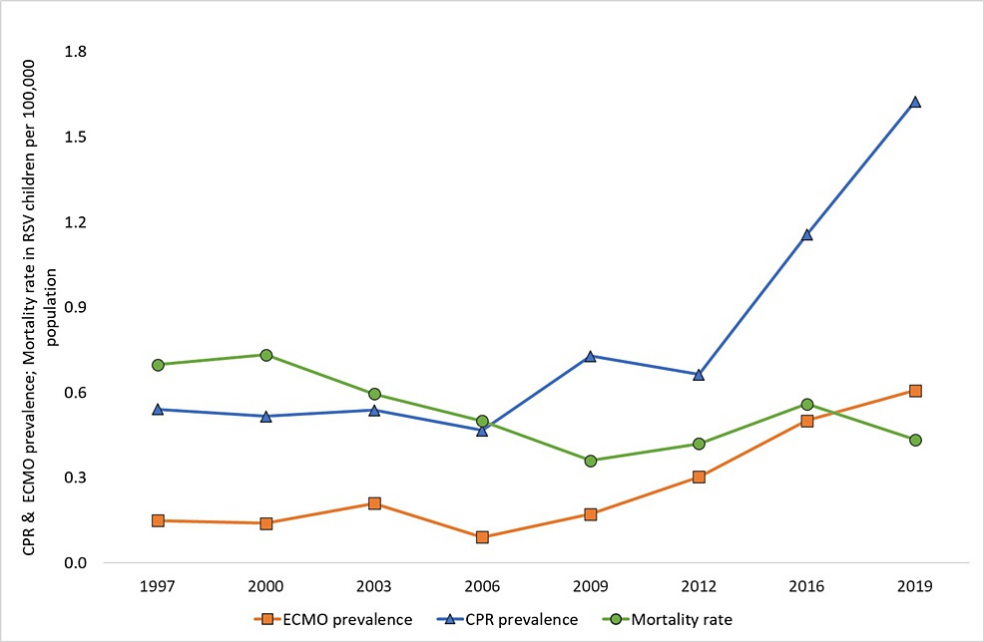
Prevalence of extracorporeal membrane oxygenation support, cardiopulmonary resuscitation, and mortality per 100,000 population among children one to 24 months with RSV infection from 1997 to 2019. CPR: cardiopulmonary resuscitation; ECMO: extracorporeal membrane oxygenation; RSV: respiratory syncytial virus

There was no trend in hospitalization rates for RSV bronchiolitis per 100,000 U.S. population during the study period. The prevalence of comorbid conditions (CCC and CHD) and the rate of use of ventilator support (NIV and IMV) in RSV children per 100,000 U.S. population increased. However, the RSV-associated hospital mortality rate per U.S. population decreased during the study period. The trend analysis results per U.S. population are presented in Figures 1, 2.

Multivariable analyses

The results of binary logistic regression analyses of demographic risk factors for non-invasive ventilation, invasive ventilation, and mortality in infants with respiratory syncytial virus infection are presented in Table 3. The adjusted odds of NIV and IMV increased during the study period compared to 1997. Meanwhile, the odds of hospital mortality decreased during the study period compared to 1997. With an increase in quartiles of MHI, the adjusted odds of NIV increased while the odds of IMV and hospital mortality decreased. The adjusted odds for gender and age group are presented in Table 3.

**Table 3:** Multivariable analysis of demographic risk factors for non-invasive ventilation, invasive ventilation, and mortality in hospitalized children one to 24 months with respiratory syncytial virus infection in the United States from 1997-2019. AUROC: area under receiver operating curve; CCC: complex chronic condition (neonatal, respiratory, and technology-dependent CCC); CHD: congenital heart disease; CI: confidential interval; MHI: median household income in quartiles

Variables	Non-invasive ventilation		Invasive ventilation		Hospital mortality	
	Adjusted Odds Ratio	95% CI	Adjusted Odds Ratio	95% CI	Adjusted Odds Ratio	95% CI
Age groups 12-24 months (ref = 1-12 months)	0.73	0.69-0.77	0.46	0.43-0.48	0.91	0.72-1.14
Female (ref = male)	0.91	0.88-0.95	0.92	0.89-0.94	1.19	1.00-1.43
MHI 2^nd^ Quartile (ref = 1^st^ quartile)	1.06	1.00-1.12	0.99	0.95-1.03	1.06	0.85-1.32
MHI 3^rd^ Quartile (ref = 1^st^ quartile)	1.19	1.13-1.25	0.92	0.88-0.96	0.88	0.69-1.13
MHI 4^th^ Quartile (ref = 1^st^ quartile)	1.56	1.48-1.65	0.91	0.87-0.96	0.56	0.41-0.76
CCC (ref = absent)	2.48	2.30-2.68	7.02	6.68-7.37	18.22	14.82-22.40
CHD (ref = absent)	1.92	1.79-2.07	3.73	3.55-3.92	6.54	5.26-8.12
Calendar year (ref = 1997)						
Calendar year 2000 (ref = 1997)	1.81	1.46-2.26	1.22	1.14-1.31	0.83	0.60-1.15
Calendar year 2003 (ref = 1997)	2.54	2.06-3.13	1.20	1.12-1.28	0.76	0.55-1.06
Calendar year 2006 (ref = 1997)	3.93	3.22-4.79	1.25	1.17-1.34	0.59	0.42-0.84
Calendar year 2009 (ref = 1997)	5.88	4.84-7.14	1.25	1.17-1.34	0.43	0.30-0.63
Calendar year 2012 (ref = 1997)	8.99	7.44-10.86	1.25	1.17-1.34	0.45	0.32-0.65
Calendar year 2016 (ref = 1997)	25.43	21.17-30.56	1.81	1.70-1.93	0.53	0.38-0.75
Calendar year 2019 (ref = 1997)	44.05	36.74-52.82	1.77	1.67-1.89	0.30	0.21-0.43
X^2^ (df)	15089 (14)		9965 (14)		1132 (14)	
p-value	<0.001		<0.001		<0.001	
Negelkerke R^2^	15.3%		7.3%		14.6%	
Brier score	0.0001		0.03		0.0009	
AUROC (95% CI)	0.802 (0.797-0.806)		0.676 (0.671-0.682)		0.793 (0.758-0.828)	

## Discussion

Our analysis of the KID revealed an increasing trend of associated comorbidities and the use of invasive and non-invasive mechanical ventilation in children one to 24 months with RSV infections in the U.S. from 1997 to 2019. RSV-associated hospitalization rates per hospital discharge increased. However, the hospitalization rates remained stable at a population level during the study period. Despite the increase in comorbidities and mechanical ventilation use, hospital mortality showed a decreasing trend. In this comprehensive epidemiologic report, we present the trend for hospital prevalence of RSV bronchiolitis and the frequency of associated comorbidities in those patients.

Demographic characteristics

Demographic characteristics of higher government insurance and a lower income quartile support the occurrence of increased viral infections in lower socioeconomic conditions and overcrowding [12]. Higher proportions of publicly insured as well as males were noted in another study from children's hospitals in children younger than 24 months with RSV infection [13]. A higher prevalence of RSV infection in infants of lower socioeconomic status was also reported in a study from the State Inpatient Database [14]. The seasonal variation in the prevalence of RSV hospitalization noted in our study has been reported previously [15]. Typically, in the U.S., the RSV seasonal epidemic begins in October, peaks in December, and ends in April [16]. The regional variation in RSV hospitalization noted in our study supports findings in a previous report [17].

Rate of hospitalization

A previous study reported that from 1980-1996, hospitalization of infants with bronchiolitis increased substantially, and it estimated that 65,000 to 100,000 children under five years of age are hospitalized annually with bronchiolitis due to RSV [18]. Another study estimated a pooled rate of 79,850 hospitalizations with RSV in infants [19]. We reported approximately 70,000 annual admissions with RSV in children under two years of age from 1997 to 2019. The pooled annual RSV hospitalization rate reported for infants from a systematic review was 19.4 per 1000 population, compared to a much lower rate (approximately six per 1000 population) we are reporting for children under two years of age [19]. However, a wide range of hospitalization rates were reported in the literature, from 8.4 to 40.8 per 1000 population [19]. Study type influenced RSV-associated hospitalization rates, with active surveillance studies having lower pooled rates than studies based on administrative claims or modeling approaches [19]. Barr et al. emphasized that both the National Institute for Health and Care Excellence (NICE) guidelines in the United Kingdom and the American Academy of Pediatrics (AAP) guidelines for bronchiolitis do not recommend routine respiratory viral testing in children suspected of having bronchiolitis [20]. This may underestimate hospitalization rates in studies using hospital discharge databases like ours.

Comorbid conditions

During our study, we observed an ascending trend in the proportion of patients with CCC and CHD among children hospitalized with RSV. In a study, Kristensen et al. utilized incidence rate ratios to investigate the association between various chronic conditions (both congenital and acquired) and the risk of hospitalization due to RSV [21]. The study's findings identified several unrecognized chronic conditions as independent risk factors for RSV hospitalization. There is a high burden of CCC in acutely ill children needing ICU admission [22]. In addition, there is an increase in the trend of the proportion of CCC in pediatric hospitalizations in general [23]. Our results are consistent with previous reports of an increase in the proportion of CCC and CHD in hospitalized patients with RSV bronchiolitis [24]. This finding has implications for both preventive strategies and resource allocations.

Ventilatory support

There was an increase in the utilization of NIV and IMV, with NIV showing a more pronounced rise in recent years. Potential reasons for the increase in NIV and IMV are the increased acuity of hospitalized patients due to milder cases being managed as outpatients. However, even when the rates are calculated per U.S. population, there was an increase in the use of NIV and IMV in children with RSV infection. An increased proportion of comorbid conditions among children hospitalized with RSV may partly account for increased utilization of invasive as well as non-invasive mechanical ventilatory support. Even after adjusting for chronic conditions, there was an increase in the utilization of ventilatory support over the years. Increased use of critical care in hospitalized patients has also been reported [25,26]. In a cross-sectional study from the Virtual Pediatric Systems database, between 2013 and 2022, there was a 4.8-fold increase in HFNC use, a 5.8-fold increase in NIV use, and a three-fold increase in the pediatric intensive care unit (PICU) admissions in children under two years with bronchiolitis [27].

Hospital mortality

All-cause mortality in infants with RSV infection from 1999 to 2019 was estimated at 2.7/100,000 U.S. age-specific population, with a linear decrease in mortality rate during the study period [28]. In our study, the all-cause hospital mortality in children aged one to 24 months with RSV infection was 0.5/100,000 U.S. population. Our study may have underestimated the mortality rate due to incomplete testing of children for RSV infection, whereas the previous study estimated the mortality rate through modeling [28].

The adjusted odds of mortality were higher for children with CCC or CHD and RSV infection in our study, similar to previous reports in children with medical complexity, resource utilization, and hospital mortality is higher [29]. In their meta-analysis, Chaw et al. reported that children with RSV and underlying CHD, particularly those with hemodynamically significant CHD, experienced more severe RSV-associated acute lower respiratory tract infections compared to those without CHD [30]. Despite the increased proportion of comorbid conditions in hospitalized children with RSV infections and the increased use of NIV and IMV, hospital mortality, overall, has decreased. Early recognition, early intervention for complications, and improved acute care may be the reasons for improved mortality despite higher acuity. The trend of increased acuity and decreased mortality in pediatric hospitalization has been reported previously [26].

Strengths and limitations

The strength of our study lies in its large population size and the inclusion of diverse hospital settings, which enhance the generalizability of the findings. However, certain limitations should be acknowledged. Studies utilizing administrative databases, such as the KID, have several limitations. The data are derived from coding and billing entries, making retrospective analyses susceptible to recall and misclassification biases. Assessing chronological relationships can also be challenging. The validity of studies using administrative databases may be questioned due to potential coding errors, such as omissions or inaccuracies. The testing rate for RSV infection over the years is lacking, which could influence the identification of RSV infections in hospitalized patients. Furthermore, certain risk factors for severe illness that may be present but cannot be identified could lead to misrepresentation in such studies.

## Conclusions

In conclusion, the hospitalization rates at a population level in the United States for RSV infection in children aged one to 24 months remained steady from 1997 to 2019, while hospital mortality rates showed a declining trend. Compared to other admissions, the hospitalization rate for RSV infection increased during the study period. There is an increased proportion of comorbid conditions and increased resource utilization in children with RSV. These findings are important for monitoring the effectiveness of preventive strategies for severe RSV infections.
